# Decentralized Personal Data Marketplaces: How Participation in a DAO Can Support the Production of Citizen-Generated Data

**DOI:** 10.3390/s22166260

**Published:** 2022-08-20

**Authors:** Mirko Zichichi, Stefano Ferretti, Víctor Rodríguez-Doncel

**Affiliations:** 1 Ontology Engineering Group, Universidad Politécnica de Madrid, 28040 Madrid, Spain; 2 Department of Pure and Applied Sciences, University of Urbino “Carlo Bo”, 61029 Urbino, Italy

**Keywords:** distributed ledger technology, decentralized file storage, distributed hash table, data marketplace, keyword-based search, citizen-generated data

## Abstract

Big Tech companies operating in a data-driven economy offer services that rely on their users’ personal data and usually store this personal information in “data silos” that prevent transparency about their use and opportunities for data sharing for public interest. In this paper, we present a solution that promotes the development of decentralized personal data marketplaces, exploiting the use of Distributed Ledger Technologies (DLTs), Decentralized File Storages (DFS) and smart contracts for storing personal data and managing access control in a decentralized way. Moreover, we focus on the issue of a lack of efficient decentralized mechanisms in DLTs and DFSs for querying a certain type of data. For this reason, we propose the use of a hypercube-structured Distributed Hash Table (DHT) on top of DLTs, organized for efficient processing of multiple keyword-based queries on the ledger data. We test our approach with the implementation of a use case regarding the creation of citizen-generated data based on direct participation and the involvement of a Decentralized Autonomous Organization (DAO). The performance evaluation demonstrates the viability of our approach for decentralized data searches, distributed authorization mechanisms and smart contract exploitation.

## 1. Introduction

Recent scandals have shown the harm that current data collection, storage and sharing practices can cause with regard to the misuse of personal data [1,2]. As the world is becoming more “smart”, so-called smart environments, of which smart cities [3] stand out the most, have in common the ability to transform data (in particular, personal data) into meaningful information needed by the liveness of the ecosystem they generate. Based on this transformation, indeed, they provide services that are becoming more and more targeted towards individuals. For instance, it is commonly known that personal information is used to recommend opportunities to individuals and to make their life easier. However, entities that control these data might not always operate with the aim of social good [4]. Many Big Tech companies rely on data collected about their users, usually storing this personal information in corporate databases, i.e., data silos, and transacting it to third parties with not enough transparency for individuals.

Meanwhile, among the many technologies used for general-purpose data management and storage, Distributed Ledger Technologies (DLTs) are rising up as powerful tools for avoiding control centralization. DLT and the realm of decentralized systems, such as Decentralized File Storages (DFS), that are emerging as solutions able to tackle the issue of obtaining large amounts of data that are not of dubious or of false origin, while providing more disintermediated processes [5,6]. DLTs, in this context, provide a new way of handling personal data, such as recording, storage and transfer. This can be carried out in combination with cryptographic schemes to ensure data confidentiality. By their decentralized nature, indeed, these technologies have the potential to make processes more democratic, transparent and efficient [7]. DLTs and DFS can support the creation of a Personal Information Management System (PIMS) based on decentralized data processing and Personal Data Stores (PDS) [8,9]. In PIMS, data access is granted in line with user policies and these ones, in a decentralized scenario, can be determined by the user via DLTs and smart contracts [8]. PIMS have been proposed by scholars [9,10,11] or companies [12] and are increasingly gaining attention from policymakers who currently consider mechanisms for regulating and advancing data intermediation services in general [7,8,13,14]. In the context of the European Union’s General Data Protection Regulation (GDPR) [15], PIMSs enforce the right of individuals to know the data collected about them and the right to transfer data to other service providers, i.e., data portability. Such features enable the process of moving the data sovereignty towards users, i.e., Self-Sovereign Identity [16], and of providing them more influence over access control, while allowing anyone else to be able to consume this data with transparency. All of this paves the way towards the use of personal data for open data markets and for social good. The ability to easily obtain personal data has the potential to create a marketplace where users are consumers and providers at the same time. By creating a common, decentralized and trustless infrastructure, such as a decentralized personal data marketplace, it will be possible for data owners and consumers to interact and collaborate in Peer-to-Peer (P2P) transactions [17,18]. This means facilitating the transactions of data between owners and consumers without the need for a trusted third-party broker, enabling liquid data markets [19].

The underlying research questions we aim to explore in this work are as follows:

- Are decentralized personal data marketplaces able to optimally support individuals’ personal data protection and portability?
- How can decentralized technologies foster a convergence between the protection of individuals’ personal data and the development of data aggregation solutions?

### Contributions

The contributions of this work include the description of a high-level solution for a decentralized personal data marketplace involving the use of DLTs, DFS and smart contracts for the creation of a PIMS: (i) data owners store their personal data in PDSs implemented using a DFS, such as the InterPlanetary File System (IPFS) [20]; (ii) this storage is complemented by the use of a DLT, which enables data integrity validation and indexing of the data, i.e., the IOTA DLT [21]; (iii) a distributed authorization mechanism enables access control of this data, thanks to the decentralized execution of immutable instructions of smart contracts implemented using the Ethereum protocol [22]; our authorization blockchain network executes access control to enact data owners’ preferences and to verify the authenticity of the claims; the nodes of such a network employ a cryptographic schema that enables the protection of data owner’s personal data (i.e., based on the use of a Threshold Proxy Re-Encryption [23]); (iv) finally, we provide a system for the search for data according to their content or meaning, relying on the use of a Distributed Hash Table (DHT) as a layer placed over the DLTs; indeed, data inserted into the DLTs and DFS is usually unstructured and no efficient decentralized mechanisms are present to query a certain kind of data; in our solution, data stored in DLT can be searched in a decentralized way through keywords stored in the DHT; the distinctive feature of our DHT network is that it is essentially a hypercube overlay structure [24], in which each node indexes objects representing specific indexes and addresses of a DLT using keywords.

Moreover, in this paper, we address a use case that assists us in describing the implementation of the decentralized personal data marketplace. Since our main driving force is facilitating the use of private data for the social good, we present a use case where citizens can use and give access to their personal data to produce new data, creating value for governments, businesses and other citizens as well. Through practices that promote a collaborative and co-creative approach to working together on the management, control and governance of the use of data, people and society can influence and shape data governance processes and can support greater social and economic equity [2,25]. By implementing this use case, we demonstrate the ability of our system to support these processes. In particular, our system allows citizens to take part in an organization for the creation of new datasets and to steer the developmental decisions through a Decentralized Autonomous Organization (DAO) [26]. Our system provides the elements of a framework for participatory data stewardship [2], being transparent, i.e., informing individuals about their data, and collaboration, i.e., enabling individuals to take action [25], at its core.

The original contributions and novelties of our work are summarized as follows:

- First, we provide a description of the implementation of a decentralized personal data marketplace, where
  - Our PDS is implemented using a DFS for storing personal data;
  - We use a DLT for providing data integrity, validation and indexing;
  - Our smart contract-based authorization system executes distributed data access control;
  - Our hypercube DHT enables a decentralized way of searching for data in DLTs.
  In particular, we provide a detailed description of the protocols behind the authorization blockchain and the hypercube DHT.
- Second, we provide the implementation of a use case for the architecture through the description of citizen-generated data creation based on direct participation. This consists of the development of a data aggregation solution through the use of a DAO, where members are citizens.
- Third, we evaluate the implementation’s performance by means of an experimental evaluation. More specifically, (i) we simulate a P2P network executing the hypercube DHT for decentralized search of data, (ii) we test the distributed data access control execution for the use case and (iii) we evaluate the smart contract implementation in terms of gas usage.

The remainder of the paper is organized as follows: Section 2 provides a background on the main concepts and technologies used, while Section 3 focuses on related work. Section 4 presents a description of the decentralized personal data marketplace architecture. Section 5 provides the description of a citizen-generated data creation use case with the intent to present our marketplace implementation. In Section 6, the evaluation of our proposal is shown and the results are discussed. Finally, Section 7 provides the concluding remarks.

## 2. Background

In this section, we introduce the main concepts and technologies involved in our work.

### 2.1. Distributed Hash Table (DHT)

A Distributed Hash Table (DHT) is a distributed infrastructure and storage system that provides the functionalities of a hash table, i.e., a data structure that efficiently maps “keys” into “values”. It consists of a P2P network of nodes that are supplied with the table data and on a routing mechanism that allows for searching for objects in the network [24]. Each node in the DHT network is responsible for part of the entire system’s keys and allows the objects mapped to the keys to be reached. In addition, each node stores a partial view of the entire network, with which it communicates for routing information. To reach nodes from one part of the network to another, a routing procedure typically traverses several nodes, approaching the destination at each hop. This type of infrastructure has been used as a key element to implement complex and decentralized services, such as Content-Addressable Networks (CANs) [27], Decentralized File Storage (DFS) [20], cooperative web caching, multicast and domain name services.

### 2.2. Decentralized File Storage (DFS)

Decentralized File Storage (DFS) is a solution for storing files as in Cloud Storage [28] but retaining the benefits of decentralization [9]. They offer higher data availability and resilience thanks to data replication. A DFS comprises a P2P network of nodes that provide storage and follow the same protocol for content storing and retrieval. In content-based addressing, contents are directly queried through the network rather than establishing a connection with a server. In order to know which DFS node in the network owns the requested contents, it is possible to rely on a DHT in charge of mapping the contents, i.e., files and directories, to the addresses of the peers owning such data. A principal example of DFS is the InterPlanetary File System (IPFS) [20], a protocol that builds a distributed file system over a P2P network. IPFS is a DFS and a protocol created for distributed environments with a focus on data resilience. The IPFS P2P network stores and shares files and directories in the form of IPFS objects that are identified by a CID (Content Identifier). The CID acts as an immutable universal identifier used to retrieve an object in the network. Only the file digest is needed, i.e., the result of a hash function applied on the data. Users that want to locate that object use this identifier as a handle. When an IPFS object is shared in the network, it is identified by the CID retrieved from the object hash, for instance a directory with a CID equal to *QmbWqxBEKC3P8tqsKc98xmWNzrzDtRLMiMPL8wBuTGsMnR*. Even if other nodes in the network try to share the same exact directory, the CID will always be the same.

### 2.3. Distributed Ledger Technology (DLT)

Distributed Ledger Technologies (DLTs) consist of networks of nodes that maintain a single ledger and follow the same protocol, including a consensus mechanism, for appending information to it. The blockchain is a type of DLT where the ledger is organized into blocks and where each block is sequentially linked to the previous one. The execution of the same protocol, i.e., source code, guarantees (most of the time) the property of being tamper-proof and not forgeable. This allows for a trust mechanism to be created without the need for third-party intermediaries [29,30].

There are different implementations of DLTs, each one with its pros and cons. In permissionless ones, anyone can take part in the consensus mechanism, while this is not true in permissioned ones. Another distinction lies in the support of smart contracts, e.g., Ethereum [22]. This feature is quite often in contrast with other key features related to the level of scalability and responsiveness of the system [31]. Conversely, some implementations are thought to provide better scalability at the expense of lacking some features. IOTA [21], for instance, implements a more scalable solution for distributing the ledger. It consists of a Layer-1 solution, while, on the other hand, Layer-2 solutions are technologies that operate on top of an underlying DLT to improve its scalability [32].

### 2.4. Smart Contract and Decentralized Autonomous Organization (DAO)

A smart contract is a new paradigm of contracts that does not completely embody the same features of a legal contract but can act as a self-managed structure able to execute code that forces agreements between two or more parts. A smart contract consists of instructions that, once distributed on the ledger, cannot be altered. Thus, the result of its execution will always be the same for all DLT nodes running the same protocol. When a smart contract is deployed on the DLT and the issuer is confident that the code embodies the intended and proper behavior (e.g., by reviewing the code), then transactions originating from that contract do not require the presence of a third party to have value [33].

Smart contracts are fundamental components of Ethereum that reside on the blockchain and are triggered by specific transactions [34]. Moreover, smart contracts can communicate with other contracts and even create new ones. The use of these contracts grants permission to build Decentralized Applications (dApps) and Decentralized Autonomous Organizations (DAOs) [6,32,35,36,37]. A DAO is a virtual entity managed by a set of interconnected smart contracts, where various actors maintain the organization state by a consensus system and are able to implement transactions, currency flows, rules and rights within the organization. Members of a DAO are able to propose options for decisions in the organization and to discuss about and vote on those through transparent mechanisms [26].

### 2.5. IOTA and Streams

In this work, we specifically refer to the IOTA DLT as a technology that uses a different paradigm for managing the ledger; however, there are many other alternatives such as Radix [38] or Nano [39]. IOTA is a DLT that allows hosts in a network to transfer immutable data among each other. In the IOTA ledger, i.e., the Tangle [21], is based on a Directed Acyclic Graph (DAG) where the vertices represent transactions and edges represent validations to previous transactions. The validation approach is thought to address two major issues of traditional blockchain-based DLTs, i.e., latency and fees. IOTA has been designed to offer fast validation, and no fees are required to add a transaction to the Tangle [40]. When a new transaction is to be issued, two previous transactions must be referenced as valid (i.e., tips selection), and then, a small amount of Proof-of-Work is performed.

An important feature offered by IOTA are the Streams [41]. Streams consist of a communication protocol that adds the functionality to emit and access encrypted message streams over the Tangle [40]. Message streams assume the form of channels, i.e., a linked list of ordered messages stored in transactions. Once a stream channel is created, only the channel author can publish encrypted messages on it. Subscribers that possess the channel encryption key (or set of keys, since each message can be encrypted using a different key) are enabled to decode messages. A channel is addressed using an “announcement link”. In other words, IOTA Streams enable users to subscribe and follow a messages stream channel, generated by some device. From a logical point of view, channels are an ordered set of messages; in fact, a channel is referenced through the link of a “starting” message.

### 2.6. Proxy Re-Encryption (PRE) and Cryptographic Threshold Schemes

Distributed systems usually store data as they are received, without further processing for confidentiality. Therefore, data can be accessed by any network participant. In order to deal with the protection of personal data, we employ in this work two cryptographic schemes, which are described in the following. Proxy Re-Encryption (PRE) is a cryptographic protocol where it is not necessary to know the recipient of the data in advance [42]. PRE is a type of public key encryption based on the figure of a proxy. A sender encrypts a plaintext with a specific public key obtaining a ciphertext. Then, the untrusted proxy transforms the ciphertext into a new ciphertext decryptable with the recipient private key, which does not have anything to do with the first public key. This operation is performed without learning anything about the underlying plaintext. This is possible using a re-encryption key generated by the sender using the recipient public key and shared with the proxy.

A Threshold Proxy Re-Encryption (TPRE) adds a layer of complexity [23]. A (t,n)-threshold scheme can be employed to share a secret among a set of *n* participants, allowing the secret to being reconstructed using any subset of *t* (with t≤n) or more fragments, but no subset of less than *t*. In a network where more than one node keeps secret fragments, a mutual consensus can be reached when *t* nodes provide the shares to a secret recipient, enabling the secret to be known by the latter. This can be used by a sender to share the re-encryption key in fragments with a network of proxies; none of the latter can obtain the whole key without the help of other t−1 proxies.

## 3. Related Works

In this section, we described the related work based on the different topics we have addressed in this work. To the best of our knowledge, no other works have developed a personal data marketplace using the same set of technologies and techniques; thus, we subdivided this section in work related to each part or parts of our proposed solution.

### 3.1. Decentralized Data Marketplace

The use of DLTs has been proposed for the implementation of data marketplaces to take advantage of the following advantages [43,44]: (i) no need to rely on third party platforms, (ii) better resilience against network partitioning and single point of failure, and (iii) privacy-preserving mechanisms [45]. Most of the related work investigated the data distribution through DLTs, focusing in particular on the use of off-chain storage based on DFS with data links referenced in DLTs [6,35,45]. Data exchange with such technologies can lead to a transparent market, where transactions between data owners and data consumers are recorded on DLTs and where smart contracts enable the self-enforcement of fair exchanges between participants and the automatic resolution of disputes [46]. In [5], the authors provided the implementation of a data marketplace based on the use of DFS for storing data and a payment protocol that exploits Ethereum smart contracts. Similarly, in [17,18], the proposed systems were based on P2P interactions and smart contracts to reach an agreement while also integrating other components such as the IOTA DLT. Lopez and Farooq [47] presented a framework for Smart Mobility Data Market in which the participants shared their data and could transact this information with another participant, as long as both parties reached an agreement. Their work focuses on the protection of individuals’ personal information, while maintaining data transparency and users’ ruled access control. Aiello et al. [35] designed IPPO, an architecture that allows users to generate and share anonymized datasets on a distributed marketplace to service providers, while monitoring the behavior of web services to discourage the most intrusive forms of tracking.

With respect to our work, these proposals build similar architectures but lack insight into decentralized access control mechanisms and or decentralized data searches.

### 3.2. Decentralized Access Control

DLTs have desirable features that make them a reliable alternative infrastructure for access-control systems. Their distributed nature solves the single point of failure problem and mitigates the concern for privacy leakage by eliminating third parties. Traditional access-control policies have been combined with DLTs: discretionary (DAC), to manage personal data “off-chain” (i.e., not directly stored in the DLT), through the access-control policy on the blockchain [48]; mandatory (MAC), to constrain the ability of a subject to access on a datum through smart contracts [9]; role-based (RBAC), for achieving cross-organizational authentication for user roles [49]; and attribute-based (ABAC), to grant or deny user requests based on the attributes of a user, an object and environment conditions [50]. Among DLT-based access-control mechanisms, Attribute-Based Encryption (ABE) [51] offers the best policy expressiveness without introducing many elements into the system infrastructure. ABE encrypts the data using a set of attributes that form a policy. Only those who have a secret key that meet the policy can decrypt the data. In [51], the authors designed a system using ABE-based access control and smart contracts to grant data access, with similar policies mechanism to our solution, while the authors of [52,53] proposed similar frameworks that combined DFS and blockchains to achieve fine-grained ABE-based access control. However, in any of the three previous cases, the secret attribute keys are issued directly by the data owner in the DLT or by a central authority.

### 3.3. Decentralized Data Search

With respect to our hypercube DHT contribution, a decentralized data search on DLT and DFS is a field that has been addressed by both scholars and developers with only a few efforts. Indeed, one of the concerns that is still open with respect to these novel technologies, is related to implementing data discovery and lookup operations in decentralized way. The Graph is one of the first protocols (actually the most used) with the aim of providing a “Decentralized Query Protocol” [32]. The Graph network consists in a Layer-2 protocol based on the use of a Service Addressable Network, i.e., a P2P network for locating nodes capable of providing a particular service such as computational work (instead of objects just as a CAN). In [54], the authors proposed a Layer-1 keyword search scheme that implements oblivious keyword search in DFS. Their protocol is based on a keyword search with authorization for maintaining privacy with retrieval requests stored as a transaction in a blockchain (i.e., Layer-1). Specifically for IPFS [20], in order to overcome the file search limitation, a generic search engine has been developed, namely “ipfs-search” [55]. This solution is rather centralized and does not escape the problem of concentration similar to the conventional web. In response to this, a decentralized solution called Siva [56] has been proposed. An inverted index of keywords is built for the published contents on IPFS and users can search through it; however, Siva is proposed as an enhancement of the IPFS public network DHT and does not feature any optimization for a keyword storage structure apart from the use of caching. Finally, a Layer-2 solution for the keyword search in DFS has been proposed in [44], where a combination of a decentralized B+Tree and HashMaps is used to index IPFS objects.

### 3.4. Decentralized Personal Data Management

The popularity of Internet of Things devices and smartphones and the associated generation of large amounts of data derived from their sensors [57] has resulted in an interest of individuals in the production and consumption of data via a data marketplace [11]. Making data, which are mostly personal, available for access and trade is expected to become a part of the data-driven digital economy [14]. In this context, we find a set of technologies referred to as Personal Information Management Systems, which help individuals reach the vision of Self-Sovereign Identity (SSI). SSI consists of the complete control of individuals’ digital identities and their personal data through decentralization. SSI has been generically implemented as a set of technological components that are deployed in decentralized environments for the purpose of providing, requesting and obtaining qualified data in order to negotiate and/or execute electronic transactions [16].

The databox, for instance, is a PDS [8,9] that must be conceived as a concept that describes a set of storing and access-control technologies enabling users to have direct control of their data. In [11,58], the databox is a platform that provides means for individuals to manage personal data and control access by other parties wishing to use their data, supporting incentives for all parties. An undirected link to this model that puts in practice the concept of SSI is the Solid project [12]. Solid has the purpose of letting users choose where their data resides and who is allowed to access and reuse it. Semantic Web technologies are used to decouple user data from the applications that use this data. The storage itself can be conceived in a different manner, while the use of Semantic Web represents to us the core element that eases data interoperability and favors reasoning over individuals’ policies. Semantic Web standards bring structure to the meaningful contents of the Web by promoting common data formats and exchange protocols, such as ontologies. The advantages consist in the fact that many ontologies are recommended by the World Wide Web Consortium (W3C) and are thus universally understood and that reasoning with the information represented using these data models is facilitated by mapping with a formal language. An example is the Open Digital Rights Language (ODRL) policy expression language. This can be used in conjunction with other standard ontologies to manage the access control to personal data in Solid [59]. Another possible approach is to program policy expression languages such as smart contracts, in order to manage control automatically [60].

## 4. Decentralized Personal Data Marketplace Architecture

In this paper, we are interested in describing the fundamentals of a decentralized personal data marketplace: (i) data marketplace because we intend to provide a system that enables data owners to benefit from the sharing of the data they own; the benefits can be purely economical but also linked to the participation to an ecosystem, e.g., sharing data for social good and research; on the other hand, we intend to provide an easier data access to data consumers, especially to the ones who do not have the resources to compete with Big Tech companies; (ii) personal data because we specifically focus on the type of data that is generated by individuals through their personal devices; thus, we assume that the role of data owner in the system is going to be engaged by individuals themselves or by some other entities on their behalf, with a strong emphasis to the concept of Self-Sovereign Identity [16]; and (iii) decentralized because we make use of several decentralized systems that help to more easily achieve a disintermediation in the process of transacting data.

**Figure 1:**
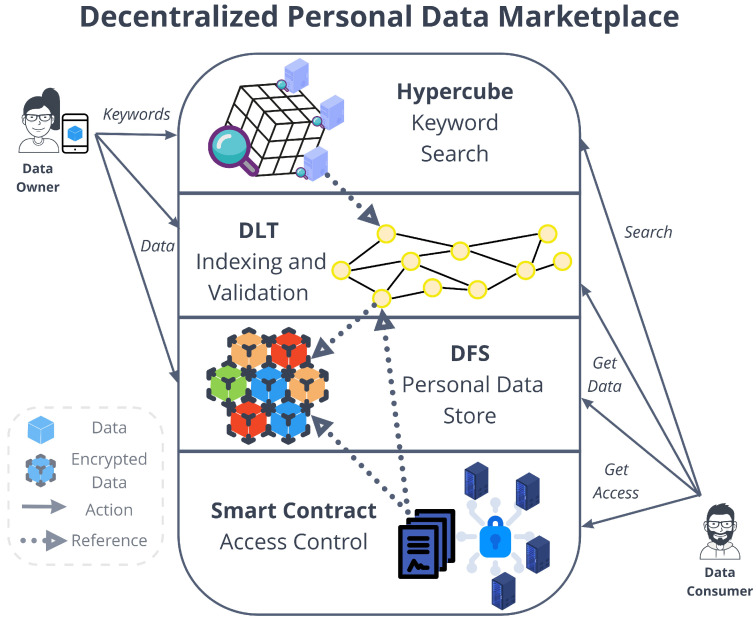
Decentralized data marketplace architecture.

In this section, we devise the marketplace architecture through a description of four pillar systems and their interactions. As shown in Figure 1, the different architectural components can be organized into four layers:

- A Decentralized File Storage (DFS) is used to store personal data in an encrypted form and to create immutable universal identifiers that directly represent the content of a piece of data. This kind of system is used to take advantage of the property of high data availability that is often taken for granted in centralized file storages.
- Smart contracts are used to provide decentralized access control mechanisms that can be leveraged by data consumers to access data retrieved from the DFS, following a policy indicated by the data owner (e.g., access through payment).
- A Distributed Ledger Technology (DLT) is used to enable the data indexing and validation. The ledger’s untamperability property makes sure that data integrity can be validated by storing data’s immutable universal identifiers, specifically in the form of hash pointers. Moreover, related pieces of data can be already linked and indexed in this layer.
- An hypercube-structured Distributed Hash Table (DHT) is used to provide a distributed mechanism for the search of data. This system is in charge of associating keywords to addresses or references stored in the DLT.

With respect to this architecture, in the following subsections, our aim is twofold: (i) to describe in detail the hypercube DHT system and (ii) to describe the interaction between all the architectural components. More specifically, we do not go into the details of all the possible configurations of the DFS, DLT and smart contracts layers, as the discussion may become too scattered and may stray away from the issues related to the decentralized personal data market.

### 4.1. DFS-Based Personal Data Store

Data generated by personal devices or third-party systems on behalf of individuals are often private in nature, but incentivizing their sharing (as opposed to keeping them locked in data silos) can be beneficial in terms of economic gain and social good. However, the main challenge is often to provide access under certain conditions that data subjects find acceptable and compliant with regulations (e.g., GDPR).

A technological solution that is opposed to centralized data silos consists of the use of DFS for storing such personal data. DFS are usually built on top of a P2P network that is freely accessible and where nodes execute the same protocol to store and retrieve data. Moreover, often at the heart of such systems, we find the provision of data replication protocols that enable a high data availability. All this means that data owners holding some data in their device can easily participate in the DFS network or reach a DFS node to store and replicate data. This use, then, makes data owners confident that their data can be retrieved by any data provider that, in turn, can participate in the network or contact a DFS node. However, to be on the safer side, data owners should incentivize DFS nodes to store and replicate their data. How to do this is beyond the scope of this paper and we refer the reader to our previous work that also investigates this topic [9].

DFSs have often built in their protocols the identification of data through immutable universal identifiers that directly represent their content in order to uniquely identify contents that are disseminated in the network. An implementation of this feature would be the use of the hash digest of a piece of data with the aim of obtaining a deterministically derived identifier. Thus, any node of the network holding the same piece of data, i.e., with exact content, can use its hash to derive its immutable universal identifier. Any other node in the network can use this id to retrieve the piece of data from other nodes and to verify its integrity through the hash.

Finally, due to the fact that data can be easily replicated in the p2p network and, thus, can be easily accessed by nodes that the data owner might be not aware of, we resort to the use of encryption as a mechanism of data protection. Such a mechanism is required both by the privacy needs of data owners and, specifically, by compliance with personal data regulations. Strong and state-of-the-art cryptographic algorithms help avoid the re-identification of such pseudonymous data, i.e., encrypted personal data, when shared in the DFS network [61].

### 4.2. Smart Contract-Based Distributed Access Control

Smart contracts are the part of the proposed architecture where access-control logic to share encrypted personal data is performed. Through dedicated smart contracts, access to data can be purchased or can be enabled directly by the owner. Access is authorized only to consumers indicated by the policies of a data owner’s contract. A policy would be for the smart contract to maintain an Access Control List (ACL) that represents the rights to access one or more pieces of data. In the rest of the paper, we focus on the application of such a policy.

According to our solution, nodes in a network that maintain a permissioned blockchain are responsible for enforcing the access rights specified in the ACLs of smart contracts. We take advantage of the high degree of trust that a blockchain provides for the data written in the ledger and, then, focus on the trust given to the nodes of this “authorization” blockchain, which must read from the ledger and follow the correct policy. If a data consumer is enlisted in the ACL, then this one is eligible to access certain data. If that is the case, then the consumer is also eligible to obtain the key used for encrypting the data in the DFS. Authorization blockchain nodes rely on ACLs to make sure that a data consumer entitled to this information can obtain such a key. For the encryption operation, we refer to a hybrid cryptographic scheme, making use of both asymmetric and symmetric keys. Generally, each piece of data is encrypted using a symmetric “content” key *k*, and then, this key is encrypted using an asymmetric keypair ($pk_{KEM}$, $sk_{KEM}$). This consists of a Key Encapsulation Mechanism (KEM) [62], in which the key is encapsulated and the capsule is distributed, instead of distributing the encrypted data.

#### 4.2.1. Access Mechanism

To ensure complete protection of the individual’s data, only the authorized recipient of personal data should obtain the key, and nodes on the authorization blockchain should not be able to exploit it. For this reason, we make use of a (t,n)-threshold scheme to share the capsule that contains the content keys among the blockchain nodes. In particular, the Threshold Proxy Re-Encryption (TPRE) scheme is employed:

- **Keypairs**—each actor creates a set of asymmetric keypairs, e.g., the data owner creates ($pk_{DO}$, $sk_{DO}$) while the data consumer creates ($pk_{DC}$, $sk_{DC}$).
- **Capsule**—a capsule is created by the data owner for each piece of data stored in the DFS. Recall that the content key *k* is used for encrypting the piece of data. Then, the result of the encryption of *k* results in the capsule.
- **Re-encryption key**—The re-encryption key $rk_{DO\to DC}$ is created by the data owner for each data consumer through the public key $pk_{DC}$.
- **Kfrags**—The data consumer divides the re-encryption key into *n* fragments following the (t,n)-threshold scheme. The single re-encryption key fragments are unique for each authorization blockchain node. We call these key fragments “kfrags” for simplicity.
- **Cfrags**—Each authorization blockchain node receives the same capsule and a unique kfrag. The capsule cannot be “opened” because it is encrypted with the data owner’s public key. Only one kfrag (or a number less than *t*) cannot be used to completely re-encrypt the capsule in such a way that the data consumer can open the capsule. The authorization blockchain node only performs a re-encryption operation that takes as input the capsule and the unique kfrags, and it outputs a new capsule fragment, “cfrag”. The data consumer requires at least *t* cfrags to reconstruct the new capsule and to decrypt it with the private key $sk_{DC}$.

The access mechanism is as follows: (i) the public key of the data consumer $pk_{DC}$ is listed in the ACL (provided in detail in Section 5); (ii) the data consumer requests the release of a cfrag to at least *t* authorization blockchain nodes using a message signed with $sk_{DC}$; (iii) upon consumer request, each node checks if the signatory $pk_{DC}$ is in the ACL through an interaction with the smart contract in the blockchain; (iv) if this is the case, then each node releases the cfrag; (v) once the data consumer obtains *t* cfrags, the capsule can be reconstructed and decrypted with $sk_{DC}$; and (vi) the decryption reveals the content key *k* needed to decrypt the desired piece of data stored in the DFS.

### 4.3. DLT Indexing and Validation

One of the main use cases of DLTs consists in data sharing due to their intrinsic property of untamperability. Once collected, in many cases, data can be stored directly on-chain, in a DLT, to validate their integrity. However, preventing the on-chain storage is a preferable solution, not only for retaining high data reads availability and better performances for data writes [9] but also because on-chain personal data are generally incompatible with data protection requirements (i.e., to guarantee personal data deletion to a data subject). Thus, our solution consists of storing personal data in a DFS and reference them in a DLT via their immutable universal identifiers, e.g., hash pointers. Moreover, due to the nature of some proposed DLTs, related pieces of data can be already linked and indexed in the ledger. That is the case of the IOTA DLT, which manages the upload of data in the form of a stream channel thanks to the Streams protocol. We refer to this DLT and this protocol to ease the description of the following parts.

#### Layer-2 Solution

**Figure 2:**
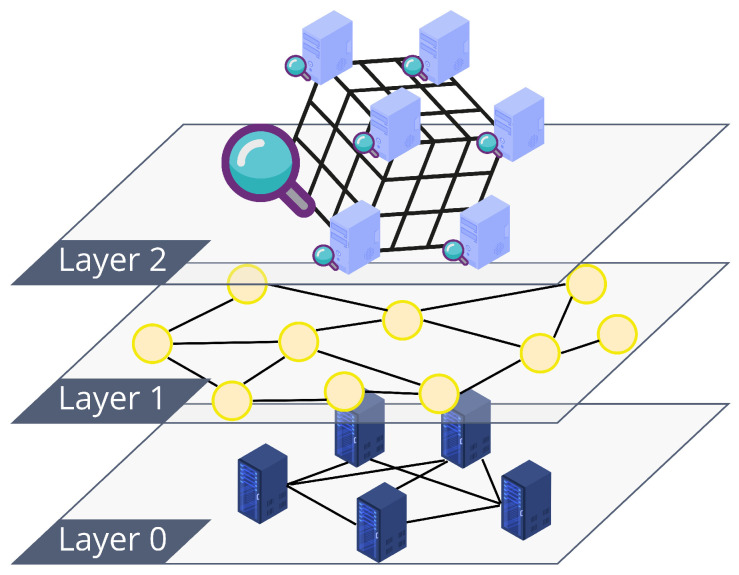
Layers in the context of DLTs. Layer zero consists of the DLT network, while Layer-1 is the set of software frameworks run by the network nodes (e.g., the ledger). Layer-2 solutions are the ones that leverage Layer-1 for other services, i.e., the hypercube DHT in our case.

While Layer-1 solutions in DLTs define the form of the ledger, its distribution, consensus mechanism and features, Layer-2 solutions are built on top of Layer-1 without changing its trust assumptions, i.e., the consensus mechanism or the structure [63,64]. Layer-2 protocols allow users to communicate through mediums external to the DLT network, reducing the transaction load on the underlying DLT. On top of the IOTA layer-1 DLT, we designed a Layer-2 solution using a DHT with the aim of facilitating the search for large amounts of data through specific keywords (Figure 2). In order to obtain information from a IOTA message within a stream channel, indeed, it is necessary to know the exact address of the message or of the channel, i.e., the announcement link. However, the announcement link of a stream channel does not provide any information related to the type and kind of messages. No mechanisms are provided by IOTA (and the majority of DLTs) for the discovery based on the content of certain data/streams channels that are available in the Tangle. This is the issue we deal with in this paper. In the remainder of this section, we describe how to surmount such limitations. In our system, every stream channel is indexed by a keyword set and then how such a keyword set is exploited to look for specific kinds of contents.

### 4.4. Hypercube-Structured DHT

Considering *O* as the set of all stream channels in IOTA, the idea is to map each object o∈O to a keyword set $K_{o}\subseteq W$, where *W* is the keyword space, i.e., the set of all keywords considered. In general, we refer to K⊆W as a keyword set that can be associated to a data content (i.e., the metadata associated to it) or a query (i.e., we are looking to some content with a specific metadata). By using a uniform hash function h:W→{0,1,…,r−1}, a keyword set *K* can be represented by the result of such a function, i.e., a string of bits *u* where the 1s are set in the positions given by one(u)={h(k)∣k∈K}. In other words, each k∈W has a fixed position in the *r*-bit string given by h(k), and that position can be associated to more than one *k* (i.e., hash collision). Then, every keyword set *K* is represented by a *r*-bit string where the positions are “activated”, i.e., are set to 1, by all the k∈K.

We use these *r*-bit strings to identify logical nodes in a DHT network, e.g., for r=4, a node id can take values such as 0100 or 1110. In particular, inspired by [24], we refer to the geometric form of the hypercube to organize the topological structure of such a DHT network. $H_{r}\left(V,E\right)$ is a *r*-dimensional hypercube, with a set of vertices *V* and a set of edges *E* connecting them. Each of the $2^{r}$ vertices represents a logical node, whilst edges are formed when two vertices differ by only one bit, e.g., 1011 and 1010 share an edge. In the network, the nodes represented by vertices that share an edge are network neighbors as well. To find out how far apart two vertices *u* and *v* are within the hypercube, the Hamming distance can be used, i.e., $\mathrm{Hamming}\left(u,v\right)=\sum_{i=0}^{r-1}\left(u_{i}⊕v_{i}\right),$, where ⊕ is the XOR operation and $u_{i}$ is the bit at the *i*-th position of the *u* string, e.g., for u=1011 and v=1010, we have Hamming(u,v)=1.

#### 4.4.1. Keyword-Based Complex Queries

In our system, contents can be discovered through queries that are based on the lookup of multiple keywords, associated with data. Such queries are processed by the DHT-based indexing scheme described in the previous section. The base idea is to associate a keyword set to each IOTA stream channel through the DHT. In particular each logical node locally stores an index table that associates a keyword set $K_{o}$ to the announcement link of an IOTA stream channel, i.e., the reference of an object *o*. Then, given a keyword set *K*, the associated *r*-bit string is used to reach the logical node responsible for *K* through a routing mechanism, in order to obtain the set of $\mathrm{objects}=\{o\in O|K_{o}⊇K\}$. For instance, with W = {*“Turin”, “Lingotto”, “Temperature”, “Celsius”*} and 1010 representing the keyword set K = {*“Turin, Temperature”*}, if u∈V is the node that is responsible for *K* because the id of *u* is equal to 1010, then *u* is in charge of maintaining a list of announcement links of IOTA stream channels containing the temperature of the city of Turin. Once that node is located, the $\mathrm{objects}=\{o\in O|K_{o}=K\}$ it stores in its index table can be returned or aggregated with other nodes’ objects. These objects consist of a list of announcement links that can be used to obtain messages from IOTA.

#### 4.4.2. Multiple Keywords Search

Our system provides two functions for making queries based on multiple keywords:

- **Pin Search**—this procedure aims at obtaining all and only the objects associated exactly with a keyword set *K*, i.e., $\left\{o\in O|K_{o}=K\right\}$. Upon request, the responsible node returns to the requester all the announcement links of the corresponding objects that it keeps in its table associated with *K*.
- **Superset Search**—this procedure is similar to the previous one, but it also searches for objects that can be described by keyword sets that include K, i.e., $\left\{o\in O|K_{o}⊇K\right\}$. Since the possible outcomes of this search can be quite large, a limit *l* is set.

For the Pin Search we need to retrieve objects only from one node, whilst for Superset Search, we need to retrieve objects from all nodes that are responsible for a Superset of *K*. Such nodes are contained in the sub-hypercube SH(S,F) induced by the node *u* responsible for *K*, where *S* includes all the nodes s∈V that “contain” *u*, i.e., $u_{i}=1\Rightarrow w_{i}=1$, while *F* includes all the edges e∈E between such nodes. Thus, during a Superset Search, the induced sub-hypercube is computed and then only nodes in such a sub-hypercube are queried using a spanning binomial tree, as described in [24] (definition 4.2). The *l* limit is a query parameter that indicates the maximum number of objects to return when traversing the spanning binomial tree.

#### 4.4.3. The Query Routing Mechanism

Queries can be injected into the system by users external to the DHT to any v∈V network node. Through a routing mechanism, the query reaches a node u∈V that is responsible for a keyword set *K*. This process is described in detail in Algorithm 1.
**Algorithm 1:** Query Routing Mechanism
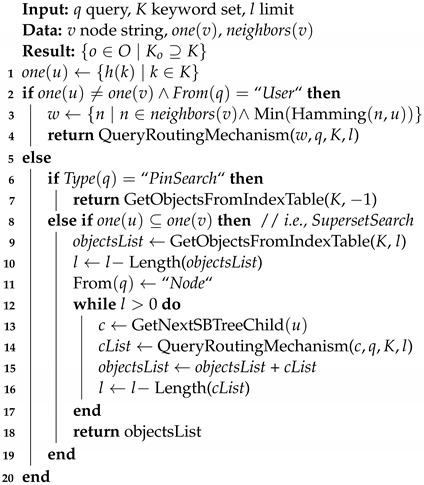


## 5. k-DaO Use Case: Participatory Data Stewardship and Citizen-Generated Data Creation

The aim of this section is to describe a possible implementation of the above architecture through a specific use case. We first describe the scenario and then we go into the details of the technical specification. With this scenario, we find ourselves in the general context of facilitating the use of privately held data for the public interest. This is in line with the vision of the European Union’s strategy on data sharing for public interest [7]. More specifically, the vision we intend to pursue with the implementation of our decentralized personal data marketplace is part of the intent to enable different stakeholders (government, businesses and citizens) to give access to and to use data transformed into non-personal form in order to create value and to make better decisions. In fact, the European Data Strategy elaborates on some points in this area, with components related to data governance and common data spaces, also by means of the Data Governance Act [14]. It enables the safe reuse of certain categories of public-sector data such as personal data.

The specific context of our scenario deals with participatory data [2] such as citizen-generated ones. Citizen-generated data, which include a range of scenarios such as participatory sensing to crowdsourced geospatial datasets, can be integrated with open data portals and, in the future, with shared data spaces. Although, to date, they are not as impactful, the aim is to increase and improve the presence of such data and to involve citizens in designing open data policy, processes and governance [65]. In most cases, citizen-generated data should be made orthogonal to the application of data protection laws and regulations, e.g., GDPR. Therefore, citizen-generated data should not contain personal data or personal data shall be appropriately anonymized or aggregated.

**Figure 3:**
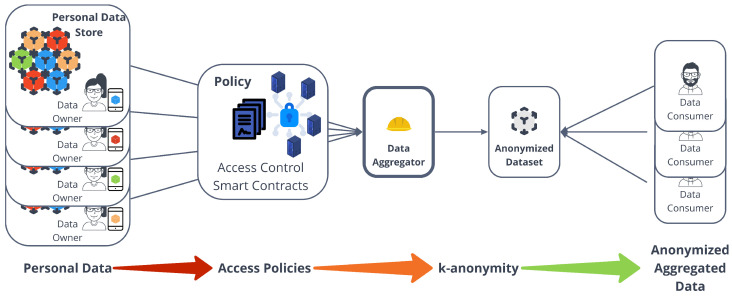
Citizen-generated data use case. Data owners store personal data in a PDS and set some access policies through smart contracts. A data aggregator accesses these data and produces an anonymized dataset in a participatory data stewardship framework. The anonymized aggregated dataset can then be accessed by other data consumers.

With this in mind, we describe the use case with the help of Figure 3. At the highest level, the flow of data is as follows: (i) citizens store and maintain their personal data in a PDS; (ii) a data aggregator undertakes the task of aggregating a specific kind of data and accesses the PDS through smart-contract access policies; (iii) the aggregator uses algorithms such as *k*-anonymity [66] to render the input personal data anonymous; and (iv) the citizen-generated anonymized aggregated dataset is published for potential data consumers. The main idea is to enable the participation of data owners in the dataset generation through a DAO. A token-based incentive for DAO members, i.e., tokenized data structures, can be used to enable participants to work together to build a curated dataset in pursuit of the instantiation of a decentralized, tokenized data marketplace [19].

We imagine a concrete scenario of citizen-generated hiking trails or pedestrian travel routes, produced using GPS-enabled smartphones using application such as Komoot or AllTrails [67]. For this scenario, one can simply consider three kinds of personal data: (i) the user’s travel trace, i.e., a set of latitude and longitude points associated with a timestamp; (ii) the user’s photos taken during the travel; and (iii) the list of nearby Bluetooth devices updated with a constant interval.

### 5.1. Anonymizing Data by Aggregation

Figure 3 shows an overview of the interaction between the main actors. Data owners (leftmost boxes) maintain personal data in a PDS implemented using IPFS [20] as DFS. These data are travel traces, photos and Bluetooth ids recorded during the data owners’ hiking sessions. Data owners also register personal data they want to share along with descriptions of what they measure, i.e., keywords in the hypercube DHT (not shown in figure; see Section 4.4). Each piece of data is then indexed in the IOTA DLT through a new stream channel for each hiking session (not shown in figure). The messages in the channel refer to data in IPFS using the CID as an immutable universal identifier. An access-control smart contract owned by the data owner (between data owners and aggregator in the figure) points to different stream channels using the associated announcement link. This smart contract is stored in a private permissioned Ethereum blockchain implemented using GoQuorum [68], i.e., the authorization blockchain. The data aggregator (at the middle of the figure) interacts with such a blockchain to request the data owners’ data in line with their policies. If it manages to access the data of at least *k* data owners, the aggregator creates a *k*-DaO with the owners in the same blockchain, in order to work in a participatory data stewardship framework [2]. The anonymized dataset must meet certain requirements; otherwise, the *k*-DaO may decide to stop production. For instance, the data aggregator should be able to perform the data aggregation, producing a dataset that presents properties of *k*-anonymity and differential privacy [69]. This dataset can then be accessed by a variety of data consumers (rightmost boxes in figure) using the same data marketplace in a process where every participant to the dataset creation is rightfully rewarded.

We now make a brief digression on what it means to apply anonymization techniques in this case. The GDPR Recital 26 states that personal data becomes anonymous if it is ‘reasonably likely’ that no identification of a natural person can be derived [13]. This is based on the fact that the anonymization of a dataset can be defined as robust on a case-by-case basis [70]. Some techniques can provide privacy guarantees and can be used to generate efficient anonymization processes but only if their application is engineered appropriately. The *k*-anonymity proposal was introduced in [66], and it is considered one of the most popular approaches for syntactic protection, i.e., each release of data must be indistinguishably related to no less than a certain number (e.g., *k*) of individuals in the population. For instance, through a generalization approach, original values are substituted with more general values, such as the date of birth generalized by removing day and month of birth. On the other hand, we find semantic techniques, i.e., when the result of an analysis carried out on a dataset is insensitive to the insertion or deletion of a tuple in the dataset. Differential privacy [69] is the main example in this case, where a dataset is released and recipients learn properties about the population as a whole but that are probably wrong for a single individual. This can be achieved for instance by adding noise to the original dataset.

### 5.2. Step Zero: Search Data on the Decentralized Marketplace

**Figure 4:**
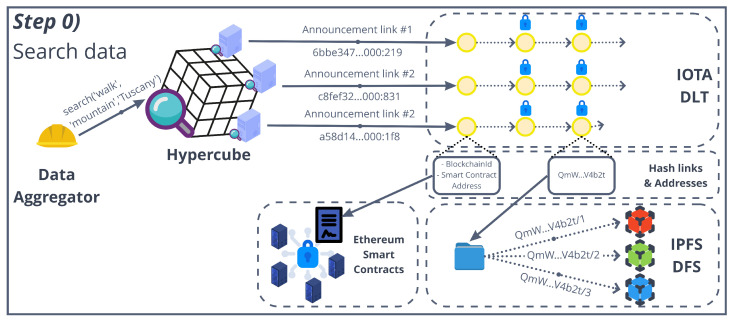
Example of searching data on the decentralized marketplace.

The first step, or “step zero”, before accessing any piece of data is the search for a specific kind of data, i.e., the data subset that a potential data consumer is interested in. This is the part where the hypercube DHT comes into play (see Section 4.4 for a detailed explanation). Figure 4 shows an example of the search of data on the decentralized marketplace. The data aggregator requests for a SuperSet Search to the hypercube, with a keyword set K = {*“walk”, “mountain”, “Tuscany”*}. The hypercube returns a set of aggregation links pointing to IOTA stream channels containing related data, e.g., *6bb3347…:219*. The first message of the stream channel is open to the marketplace users and includes information that points to the smart contract used for the access control. The information includes the identifier of the authorization blockchain network and the smart contract address. Each subsequent channel’s message includes information to the data themselves, i.e., hash links in the form of IPFS CIDs. In particular, each message stores the CID of a IPFS directory that stores the location data, photo and Bluetooth ids in a specific timestamp, e.g., *QmW...V4b2t* is the CID of the directory and *QmW...V4b2t/1* contains a location point and timestamp. Of course, the data are encrypted; hence, the content of the IPFS data are not meaningful at this point. The next step, thus, is to gain access to the content key used for the encryption.

### 5.3. Smart Contracts Implementing the Distributed Access Control

**Figure 5:**
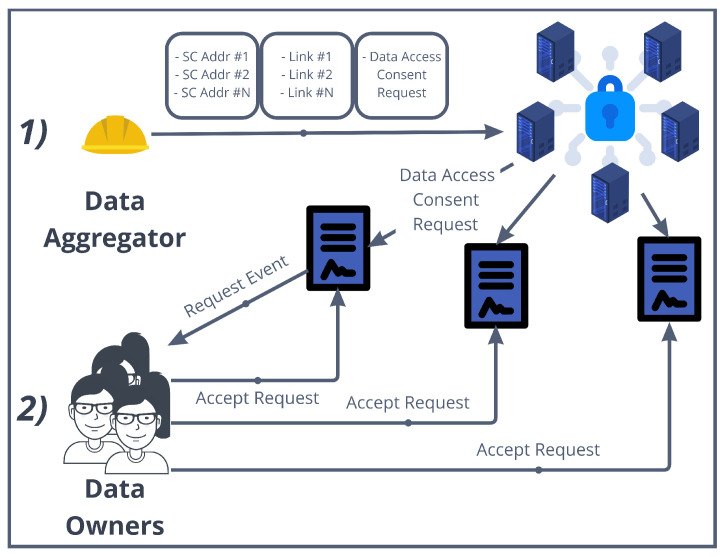
Example of the distributed access control where a data aggregator requests access to the data of some data owners.

**Figure 6:**
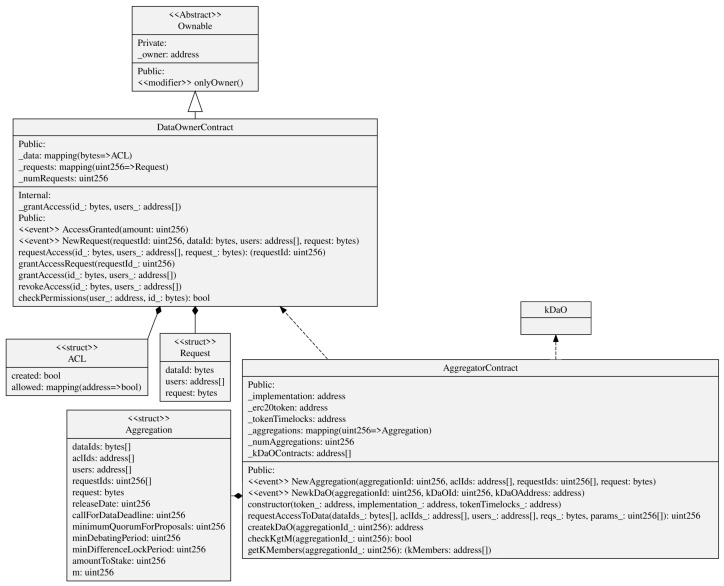
UML Class Diagram of *DataOwnerContract* and *AggregationContract*. Some classes, attributes and methods have been removed to render the diagram clearer.

As seen in Section 4.2, the interesting aspect of smart contracts is that an algorithm executed in a decentralized manner enables two parties, i.e., data owner and aggregator, to reach an agreement in the transaction of the data. This not only increases the disintermediation in such a process but also leaves traces to be later audited and provides incentives to all the actors to correctly behave. Figure 5 graphically shows the process of the data aggregator accessing data owner’s data, while Figure 6 shows the UML Class Diagram of the smart contract implementations we discuss in this subsection.

- Each data owner has previously deployed a *DataOwnerContract* in the authorization blockchain. The data aggregator too has previously deployed an *AggregatorContract*.
- In the “step zero”, the aggregator has obtained a list of *DataOwnerContract* addresses that point to IOTA stream channels through as many announcement links. Then, they produce a data access consent request in a string form for such data (the use of standardized models for the consent request, such as W3C recommended ontologies, is left as future work).
- The aggregator gives these three pieces of information as inputs to the *requestAccessToData()* method in the *AggregatorContract()*, together with a series of parameters needed for the *k*-DaO (shown in the next subsection). This method implements, in only one blockchain transaction, the request to access data for each *DataOwnerContract* found as input. In particular, the method *requestAccess()* is invoked for each *DataOwnerContract*, with the associated announcement link and request as input (Figure 6 shows *id_* as parameter representing the link and an array of addresses *users* for representing the Ethereum accounts that will be granted access).
- A *NewRequest* event will reach each data owner. This one decides to consent to the access to data based on the data access consent request received through the event. If so, the data owner invokes the *grantAccessRequest()* method in the *DataOwnerContract*.
- Among the parameters set in *requestAccessToData()*, *m* was set as the minimum number of members needed to create the *k*-DaO and to start the data aggregation process. It is also the minimum number of participants required to provide “reasonable” anonymity.
- The aggregator uses the *checkKgtM()* method to check if the number *k* of data owners that granted the access to their data is greather than *m*. When this happens, the aggregator can create the *k*-DaO through the *createkDaO()* method that instantiates a new *kDaO* contract.
- The aggregator can now access all content keys for the decryption of all the data owners’ data through the authorization blockchain nodes, as described in Section 4.2.1.

### 5.4. Smart Contracts Implementing the *k*-DaO

**Figure 7:**
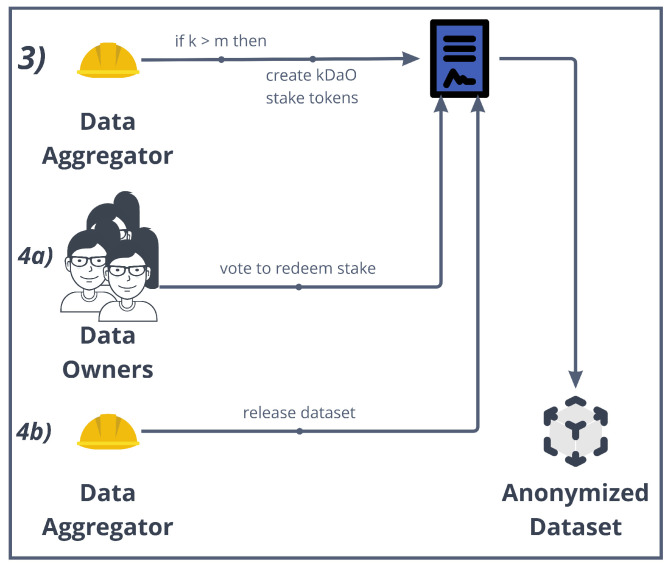
Example of the anonymized dataset creation and DAO voting.

**Figure 8:**
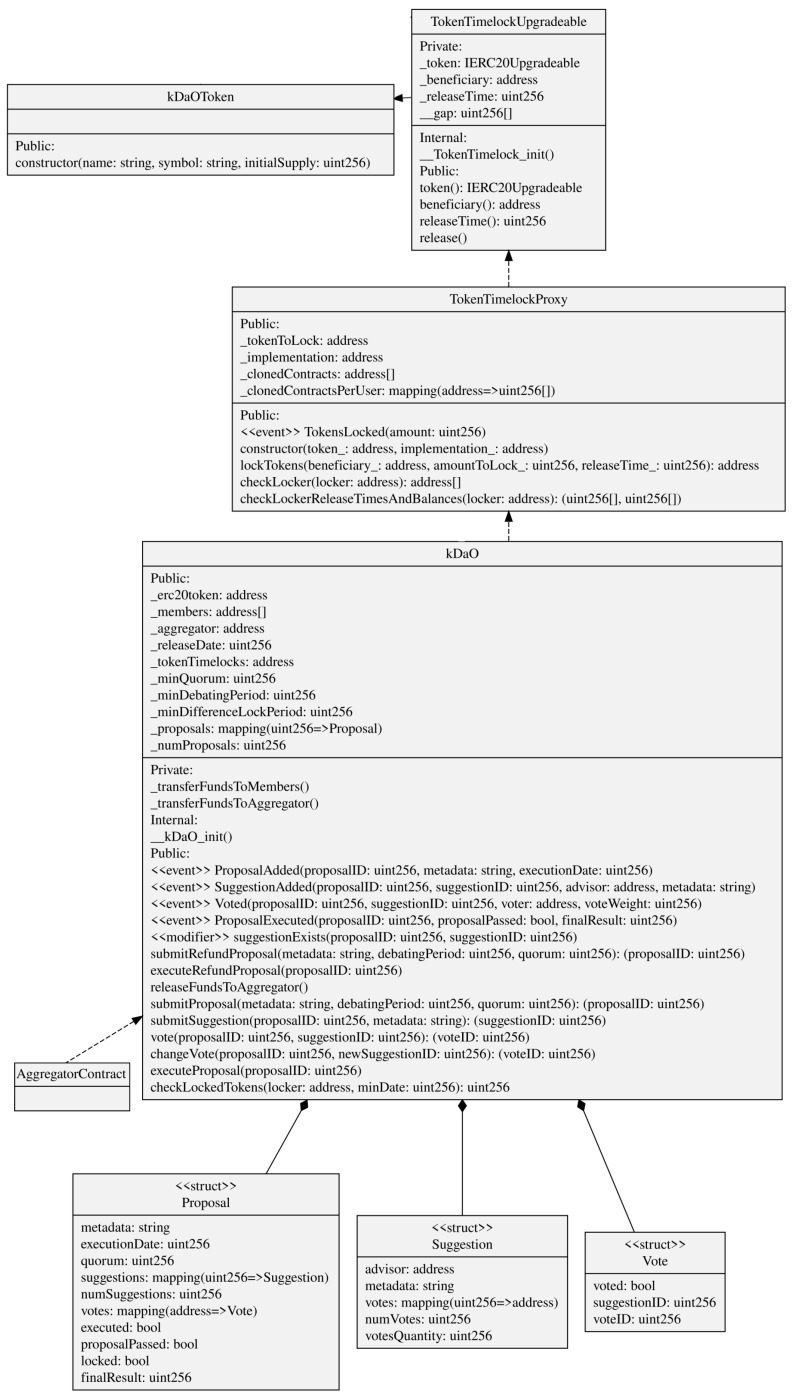
UML Class Diagram of *kDaO*, *TokenTimelockProxy*, *TokenTimelockUpgradeable* and *kDaOToken*. Some classes, attributes and methods have been removed to render the diagram clearer.

The *k*-DaO is DAO composed by the *k* Data Owners that grant access to their data to the data aggregator. Simply put, the aggregator stakes a safety deposit, and the DAO is used to start at any moment a vote to redeem this stake. The rationale behind it is to limit aggregator’s malicious behavior. Not only for this but also if the creation process of the anonymized dataset involves a more complex case of curated dataset (e.g., OpenStreetMap [71]), then DAO members can make new proposals and add suggestions to vote in order to steer the development of the dataset generation. Figure 7 graphically shows the process of *k*-DaO creation and voting, while Figure 8 shows the UML Class Diagram of the smart contract implementations we discuss in this subsection.

- A *kDaO* contract is created for each aggregation process. The *AggregationContract* acts as a contract factory but implementing a proxy pattern (EIP-1167 Minimal Proxy [72]). Instead of deploying a new contract each time such as in the factory pattern, this implementation clones an already deployed contract functionalities by delegating all methods invocation to it.
- Some DAO parameters were already set up during the request data access process, such as the amount the aggregator stakes. When the *kDaO* contract is created, a transfer of an amount of ERC20 tokens [73], i.e., the *kDaOToken*, is performed automatically from the aggregator account to the *kDaO* contract. At the end of the aggregation process, the aggregator can redeem this stake if all operations have been successful.
- *k*-DaO members can call for a vote and then decide on a proposal. Any member can make a proposal using the *submitProposal()* method, and for that proposal, all members can submit a suggestion using *submitSuggestion()*. Then, all members vote on a suggestion regarding that proposal. For instance, a proposal could be to “Change anonymization technique”, and some suggestions could be “Differential privacy” or “*k*-anonymity”. Each proposal has their own debate period and any member can invoke *vote()* to vote for a suggestion within that time period. After the debate period, the method *executeProposal()* counts the votes, if *minQuorum* is reached, then it stores the result and possibly enacts a specific procedure.
- Indeed, any extension of the previous voting smart contract can be developed to allow for a decision taken to directly enact an operation to be executed on-chain. In this case, *submitRefundProposal()* specifically starts a vote to take the data aggregator’s staked amount and to redistribute it to all members. In this case, *executeProposal()* would subdivide the staked amount to all the members if the proposal is passed.
- The *kDaOToken* is central in the DAO, as it also allows members to vote. Indeed, a member vote weight is proportional to the amount of tokens locked until a date that comes after the debate period ends. This is performed in order to avoid malicious data owners unreasonably voting to redeem the aggregator stake. A *TokenTimelockUpgreadable* is used for each token lock. This is created using the proxy pattern as well.

### 5.5. Anonymized Aggregated Dataset

Finally, the work of the aggregator comes to produce new data in the form of anonymized aggregated data, providing anonymity by design. Multiple configurations of aggregated data can be produced, if stated earlier. Additionally, some kind of proof can be implemented for measuring the exact quantity of data used from each subject’s dataset, e.g., storing in the *kDaO* contract the root of a Merkle tree that contains all the data pieces hashes used as leaves; then, *k*-DAO members can validate it by requesting (off-chain) leaves to the aggregator.

For the sake of the citizen-generated data use case, the result of the whole process is stored in an open data platform. If needed, some data, such as the participants list, can be shown upon request, but it is not public, since the authorization blockchain is a private permissioned one. In other cases, the resulting dataset can be encrypted, uploaded in IPFS and then referenced in new stream channels. In this case, the dataset is treated as all the other kinds of data in the marketplace and data consumers can access to it through a *DataOwnerContract* owned by the aggregator. In this case, some kind of royalties can be transferred directly to the *k*-DaO members, where the payment is proportional to the contribution produced by each participant, e.g., aggregator=55%, $\mathrm{data}\;{\mathrm{owner}}_{1}=20\%$, $\mathrm{data}\;{\mathrm{owner}}_{2}=10\%$, $\mathrm{data}\;{\mathrm{owner}}_{3}=15\%$.

## 6. Performance Evaluation

Based on the above *k*-DaO use case, we conducted the performance evaluation in three stages: (i) in the first stage, we simulated a DHT network implementing the hypercube queries of the use case’s “step zero”, in order to test the average steps necessary to reach all nodes; (ii) in the second stage, we set up a local permissioned authorization blockchain to test the distributed access control in use case’s steps one and two; and (iii) in the third stage, we evaluate the implementation of all smart contracts by measuring the gas usage.

In this work, we lack an analysis of the performances for storing and retrieving data from IOTA and IPFS. However, we dealt with these aspects in previous work, testing out specifically the storing of personal data such as location data and photos, i.e., testing IOTA [74] and DFS including IPFS [75]. We refer the reader to these two studies. Moreover, being separate systems, the latency in performing operations are added up one another. Meaning that a data aggregator first needs to obtain the content key from the authorization blockchain (evaluated in this work) and then operate with IOTA or IPFS.

The decentralized personal data marketplace component implementation can be found as an open source code on Github [76,77,78,79].

### 6.1. Hypercube DHT Simulation

We conducted a simulation assessment using PeerSim, a simulation environment developed to build P2P networks using extensible and pluggable components [80,81]. Once the hypercube-structured DHT was designed and implemented for multiple keyword search Section 4.4.2, we focused on studying the efficiency of the routing mechanism. The simulation implementation and the tests data can be found as open source code in [82]. Below are the main results obtained.

#### 6.1.1. Tests Setup

Several tests were carried out assuming different scenarios in which the network consisted of a variable number of nodes and stored a variable number of objects. In order to evaluate Pin Search and Superset Search, tests were carried out on different sizes of the hypercube. Specifically, the number of nodes varied from 128 (r=7) up to 8192 (r=13). Then, for each dimension *r*, a different number of randomly created keyword objects, i.e., IOTA announcement links, was inserted in the DHT. The number of objects taken into consideration varies from 100, 1000 and finally 10,000.

#### 6.1.2. Results

Given the nature of the tests, i.e., a simulated network, we considered the number of hops required for each new query as a parameter to be evaluated. A hop occurs when a query message is passed from one DHT node to the next. The query keyword sets were randomly generated, and the starting node was randomly chosen. For each type of test, 50 repetitions were performed, and then, the average results were calculated. For the Superset search, the limit value was set to l=10 objects.

##### Pin Search

**Figure 9:**
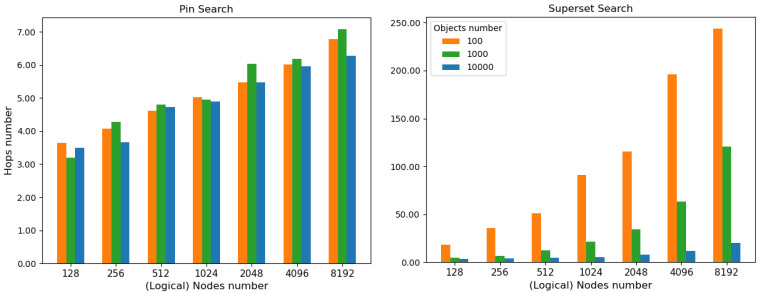
Number of hops on average for the Pin Search (**left**) and Superset Search (**right**).

As shown in Table 1 and Figure 9 (left), the number of hops required to transmit a message from the source node to the destination node increases as the hypercube dimension increases, i.e., nodes number. The average number of hops increases from about 3.5 for 128 nodes (r=7) to about 6.72 for 8192 nodes (r=13). This behavior can be explained by the fact that, by increasing the hypercube dimension, the path that a message must take before reaching its destination is automatically enlarged. The number of objects in the testbed does not affect the final outcome, since the path to reach the target node only follows the rationale of the hypercube and does not depend on the number of keyword object associations stored in the DHT.

##### Superset Search

The tests performed on the Superset Search present results with dissimilar values with respect to the previous case (Table 2 and Figure 9 (right)). At a first glance, in fact, those apparently anomalous values stand out, corresponding to a high number of hops between nodes, which decreases with the referenced object number. With a low number of objects referenced in the DHT, there are a high average number of hops needed to satisfy the Superset search. This phenomenon can be explained by the fact that the Superset search traverses the spanning binomial tree of the sub-hypercube induced by the node responsible for the keyword set, until it finds the number of objects indicated by the limit, i.e., l=10. Hence, in a network with many nodes and few objects, the query might take longer to reach that limit because many nodes are “empty”, i.e., do not reference any object. Considering the case of 4096 nodes (r=12) and 10,000 objects, in a Pin search, 5.96 hops are required, on average. In a Superset search, other 11.92−5.96=5.96 hops are needed to reach other nodes containing other results of the superset search, until the limit *l* is reached. If objects were uniformly distributed, the total number of nodes requested to return objects would have dropped to 4 nodes because each node would have maintained $\frac{10,000}{4096}=2.44$ object references on average and l=10(≅4×2.44).

#### 6.1.3. Discussion

The results obtained confirm what was expected due to the hypercube structure of the network: the Pin Search number of hops are of the order of the logarithm of the hypercube logical node number, i.e., log(n)=r. In particular, on average, they are equal to $\frac{\log(n)}{2}=\frac{r}{2}$. For what concerns the Superset Search number of hops, on average, it is equal to $\frac{\log(n)}{2}+l$, where *l* is the limit of the number of nodes in the sub-hypercube to reach.

These results show the goodness of the solution in the trade-off between memory space and response time. In traditional DLTs, such as Ethereum and IOTA, searching for a datum in a transaction means traversing all the “transaction sea” in the ledger, and for this reason, the current solution is to use centralized “DLT explorers” [83]. On the other hand, in the case of sharded DLTs, the proposed solution could become a Layer-1 protocol to search the data between many shards.

Finally, while in this study we focused on DLTs as the underlying data storage, it is worth mentioning that, due to the origins of the hypercube proposal [24], DFS systems can perfectly fit with such architecture, since most of them are based on DHT already. Indeed, the implementation of the hypercube for keywords search in IPFS is a matter of future work.

### 6.2. Authorization Blockchain Performances

In this subsection, we present the methodology and results of the performance evaluation we carried out for the authorization blockchain. We deployed all the smart contracts in a local permissioned Ethereum blockchain, using the Consensys GoQuorum implementation [68]. ConsenSys Quorum is an open-source protocol layer with the aim of building Ethereum compatible environments for enterprises. Supporting the Ethereum protocol means the possibility to execute smart contracts compiled from Solidity. Moreover, it is composed of a suite of different technologies, among which we find GoQuorum, a fork of the Ethereum node implementation in Golang. The rationale behind this choice is to be able to implement private smart contracts and transactions for protecting personal data stored on-chain by the data owners, a feature that GoQuorum supports.

We have already tested some implementations of the authorization blockchain in [9], making a comparison between two different cryptographic methods for key distribution using two open source library implementations. In this work, we test our implementation of the TPRE Umbral protocol [23], openly available as source code [77]. This is executed by the authorization blockchain nodes and thus integrated with the GoQuorum software. The client software and the smart contracts implementation is open source too and can be found in [84].

#### 6.2.1. Test Setup

During the test, we used the Istanbul Byzantine Fault-Tolerant (IBFT) consensus mechanism: each block requires multiple rounds of voting by the set of validators (>66%), recorded as a collection of signatures on the block [68]. During the tests, four validator nodes were deployed to create the base blockchain network. Each validator node executes the consensus mechanism with parameter values set up following the recommendations in [68], e.g., minimum inter-block validation time is set to 1 s. Moreover, these nodes also execute the TPRE service. One non-validator node is used to expose the APIs for external clients to interact with the blockchain. Several client nodes are created to interact with these APIs, which in turn disseminate transactions within the network [85]. The network was run on a server with a 10 cores Intel Xeon CPU and 8 GB of DDR4 RAM.

In the following, we evaluate this set of operations that implement the scenario shown in Section 5.3.

1. **Request Access**—this operation is executed by the data aggregator and consists of only one method invocation, i.e., the *requestAccessToData()* method in the *AggregatorContract*; we recall that this method requests access to data for each *DataConsumerContract* given as input.
2. **Grant Access**—this operation is executed by each data owner by invoking the *grantAccessRequest()* from their own *DataConsumerContract()*; this will store the aggregator public key $pk_{DA}$ in the smart contract ACL.
3. **Create KFrags**—this operation includes three subsequent steps; first, the owner generates a new set of *n* kfrags using the data aggregator’s $pk_{DA}$ (as described in Section 4.2.1); then, the owner sends a kfrag each to the *n* authorization blockchain nodes; finally, the owner requests to the *n* nodes the creation of a cfrag using the kfrag just got (the capsule for the piece of data interested was sent in a pre-processing step, not accounted for the measuring).
4. **Get CFrags**—the last operation is executed by the data aggregator to obtain access to the content key; the aggregator first sign a challenge-response message using the secret key $sk_{DA}$ associated to the $pk_{DA}$; then, the aggregator sends a Get CFrag request to *k* authorization blockchain nodes using the signed message; and each node validates the signature and check if $pk_{DA}$ is in the associated ACL in the *DataConsumerContract*, and if so, each node returns a cfrag to the data aggregator.

#### 6.2.2. Results

We recall that *n* is the number of validator/authorization blockchain nodes and was set to 4. We consider a round of operations the successful execution of the above described operations in order. The independent variables tested were the *threshold t*, from 1 to 4, and the *number of data owners k*, from 10 to 80 with an increase of 10 each time. We tested all the combinations of independent variables 3 times; then, we averaged the results. In each test, we initiated the round of operations 10 times for each data owner, with an interval of 3000 ms on average (value given by a Poisson Process with a mean of 3000 ms). This implies that, if overall, the set of operations lasted more than 3000 ms to be executed, probably another one was launched in parallel. This is for each data owner. The dependent metrics we measured with the tests are the *latency*, for a response to an operation, and the system *throughput*, i.e., the number of rounds of operations per second.

##### Round of Operations

**Figure 10:**
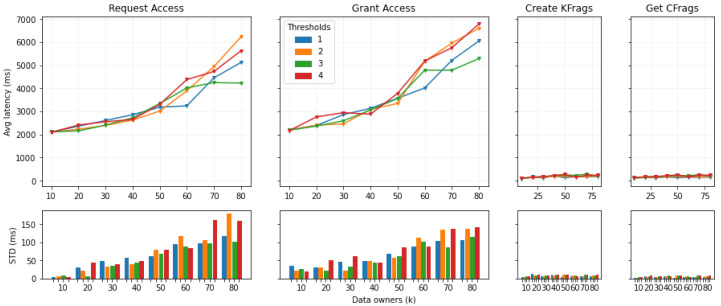
Average response latency and standard deviation for each operation in a round, varying the threshold from 1 to 4 and data owners from 10 to 80.

Figure 10 shows the average response latency and standard deviation for each operation in a round. The first result that stands out is the large difference in latency between the Request Access and Grant Access operations and the Create KFrags and Get CFrags operations. This is due to the fact that the first two operations involve writing in the authorization blockchain’s ledger. Thus, we can already see the impact of the blockchain in the overall system response latency.

As can be seen, in general, the *t* value does not affect the results greatly. On the other hand, as expected, the *k* value that represents the number of data owners is the key factor. A slow but constant increase in the round response latency happens between 10 and 40 owners, starting from 2 s latency to 3, for both Request Access and Grant Access operations. After 40 owners, the latency increases faster per number of owners. This seems to be correlated to the fact that a new round is started on average each 3 s for each data owner. Thus, if the round takes approximately more than 3 s, as from k=50 onward, many more operations start to be executed in parallel. The increase in such parallel executions seems to increase the response latency overall.

While the blockchain writing-dependent operations are in the order of the thousand milliseconds, i.e., seconds, the KFrags and CFrags operations are in the order of the hundreds and can be better analyzed using Table 3.

In both cases, we can see a direct correlation of response latency with both the *t* and *k* values. With k=10, latency values for the Create Kfrag operation are around 90 ms, while those for the Get CFrag operation are around 110 ms. With k=80, the values more or less double.

##### System Throughput

**Figure 11:**
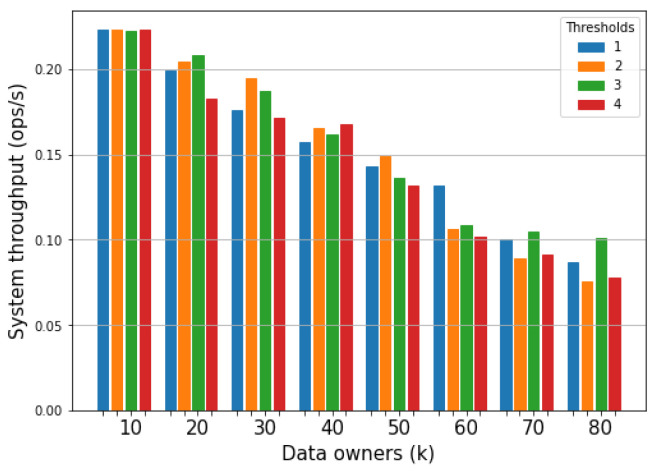
System throughput considering a round as a single operation, i.e., aggregating the results for each single operation, while varying *t* and *k*.

Figure 11 shows the results obtained when considering the round as a single operation, i.e., aggregating the results for each single operation. The figure thus shows the number of rounds per seconds, i.e., ops/s. The throughput results in more than 0.2 ops/s for the number of owners k=10 and linearly decreases with the increase in *k*. With k=80, we have on average a throughput of ∼0.07 ops/s. In this case as well, we can notice how the influence of *t* is almost irrelevant. As we have seen before, *t* influences greatly the Create Kfrag and Get CFrag operations, but these two, overall, slightly increase the round response latency with respect to the Request Access and Grant Access operations. Indeed, here too, we can see the effect of the blockchain execution in delaying the response time.

##### Threshold Number

**Figure 12:**
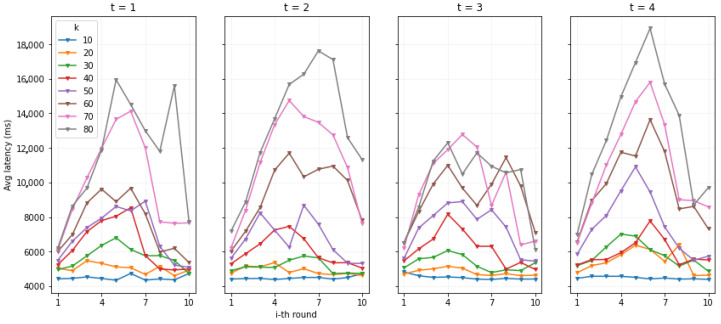
Average response latency when increasing the threshold *t* value and the number of owners *k* for each *i*-th round.

Figure 12 shows the results when increasing the *t* value and the number of owners *k* for each *i*-th round, i.e., it shows the performances for each subsequent round instead of aggregating all rounds through their mean. In this case, the results shown confirm that the increase in *t* does not influence much to the overall response delay. However, this temporal point of view shows the accumulation of delay in the response time when increasing *k*. We can see, for instance, that up to k=30 each *i*-th round has more or less the same average latency. When increasing *k*, however, the latency of rounds in the middle spikes upwards, due to the accumulation of operations to perform, and then returns to a relatively normal value in the last rounds (i.e., 9-th and 10-th).

#### 6.2.3. Discussion

Limited to the scenario we tested, it seems that a number of data owners around 30 and 40 induces the best ratio of completed rounds to response latency time. With this workload, the system can fulfill around 0.17 rounds per seconds. Overall, we can observe how the writing in the blockchain greatly impacts the whole system performance and that the number of requests related only to the TPRE operations can still scale to a larger number of data owners.

In reality, the interaction of owners with the system may be much slower, making the overall round latency increase but, at the same time, diminishing the system workload. We can imagine that the *NewRequest* event triggered by the *requestAccess()* method is shown to the data owner through a smartphone notification, thus requiring seconds, if not hours, to be read and accepted. In this context, the use of semantic web-based policy languages to express rich rules for consent and data requests could be useful in automating (and thus speeding up) this process [59]. This is left as future work.

Nonetheless, we argue that the results show the viability of our approach, especially having the possibility to tweak the authorization blockchain parameters and node hardware configuration. Moreover, the good response of the TPRE implementation gives reason to believe that, by moving this module to another blockchain that supports smart contracts but provides better latency, even improved outcomes can be achieved.

### 6.3. Smart Contract Gas Usage

Our focus is now on the execution of the smart contracts that we described in the use case Section 5, with regards to steps 1 to 4. In Ethereum, the *gas* is a unit that measures the amount of computational effort needed to execute operations. Thus, the higher the gas usage for a method, the more intense the computation of a blockchain node to execute the method’s instructions. In Table 4, we provide the execution cost for the main methods in terms of gas usage.

We start from the analysis of the gas usage of the transfer() method of the kDaOToken contract. This acts as a reference point, as this method is one of the most invoked ones in the Ethereum public permissionless blockchain, because it consists of the standard implementation of the ERC20 token. The associated gas usage of ∼52k can be relatively considered cheap, and it helps to give a measure of comparison. The *DataOwnerContract*’s methods can, then, be considered relatively cheap in comparison. This result is needed because these methods are executed many times. The method *requestAccess()* is the one with the highest gas usage because it takes as input several parameters, i.e., IOTA announcement link, variable list of Ethereum accounts, a string for the request.

The *AggregatorContract*’s method *requestAccessToData()* has an high gas usage, i.e., ∼700k, because it interacts with several other contracts on-chain. This usage value represents a request made to other two smart contracts. In general, the gas usage in this case increases linearly with the number of contracts to make the request to. The *createkDaO()* method is cheaper because it only reads from those smart contracts. However, the gas usage is high because it deploys a new contract, i.e., the *kDaO* one, using the proxy pattern. By using the EIP-1167 Minimal Proxy pattern [72] instead of a standard factory pattern, this method only uses ∼447k gas units instead of ∼2840k.

In the *kDaO* contract, the *submitProposal()* method is used to submit a generic proposal and uses less gas than the *submitRefundProposal()* because the latter executes two more operations, i.e., submits two proposals “refund” and “not-refund”. The *vote()* and *changeVote()* methods have slightly higher gas usages because of the check of the locked tokens. The *lockTokens()* method in the *TokenTimelockProxy* contract for locking a certain amount of *kDaOTokens* is expensive in terms of gas usage, i.e., ∼256k, because it also deploys a new contract using the proxy pattern. However, also in this case, there are savings compared with the factory pattern, that requires ∼1037k gas units.

Generally speaking, the methods that are executed the most do not appear to be a concern for their execution in a private permissioned blockchain environment.

## 7. Conclusions

In this paper, we have described the architecture of a decentralized personal data marketplace and provided an implementation based on Distributed Ledger Technologies (DLTs), Decentralized File Storages (DFS) and smart contracts. Data are stored in Personal Data Stores (PDS) and then accessed through an authorization blockchain using a Threshold Proxy Re-Encryption (TPRE) schema. Moreover, we have provided a Layer-2 solution based on the use of an hypercube-structured Distributed Hash Table (DHT), with the aim of facilitating the retrieval of large amounts of data using specific keywords. We focused specifically on retrieving data stored in IOTA stream channel messages. We discussed a use case for participation in the creation of citizen-generated data with the aim of describing our implementation and of validating it against a real-world scenario. The proposal validation then continued with a performance evaluation divided in three steps: (i) hypercube DHT simulation, (ii) distributed authorization testing and (iii) smart contract gas usage.

The solution we provided for the hypercube DHT consists of a decentralized system that provides an efficient routing mechanism based on keyword sets. The simulation analysis shows that searching for an object with an exact keyword set requires on average $\frac{\log(n)}{2}$ hops, where *n* is the number of logical nodes of the hypercube. This solution presents an efficient trade-off between memory space and response time, thus making a first contribution towards the creation of a system that allows complex queries on DLT.

The distributed authorization is implemented using the GoQuorum permissioned blockchain, a set of smart contracts for implementing data owner’s policies and the TPRE cryptographic schema for distributing the keys that decrypt data. The results show that writing on the blockchain represents a bottleneck, but that the citizen-generated data use case implementation is viable. Moreover, the results beyond the ledger writing part gives good reason to believe that a similar approach can be easily implemented in more performing blockchains with much better results.

Smart contracts that implement access control and DAO operations have adequate gas usage. The use of patterns such as the Minimal Proxy pattern helps to reduce the gas usage of some contract methods.

Finally, for future work, we are preparing the deployment of such a decentralized marketplace in larger networks, formed by more performing nodes. This will allow us to better test the influence of the network transmission and the system scalability. Moreover, we will focus on the integration of richer policy expression languages for managing personal data access control, adding a layer of policy declaration and reasoning on top of smart contracts.

## Figures and Tables

**Table 1:** Pin Search number of hops.

Nodes Number	Average			Standard Deviation			Confidence Interval (95%)		
	100	1000	10,000	100	1000	10,000	100	1000	10,000
**128**	3.64	3.2	3.5	1.33	1.32	1.12	(3.2, 4.0)	(2.8, 3.5)	(3.1, 3.8)
**256**	4.08	4.28	3.66	1.45	1.48	1.31	(3.6, 4.4)	(3.8, 4.6)	(3.2, 4.0)
**512**	4.62	4.8	4.72	1.57	1.70	1.24	(4.1, 5.0)	(4.3, 5.2)	(4.3, 5.0)
**1024**	5.02	4.96	4.9	1.68	1.67	1.69	(4.5, 5.4)	(4.4, 5.4)	(4.4, 5.3)
**2048**	5.48	6.04	5.48	1.76	1.85	1.69	(4.9, 5.9)	(5.5, 6.5)	(5.0, 5.9)
**4096**	6.02	6.18	5.96	1.55	1.61	1.62	(5.5, 6.4)	(5.7, 6.6)	(5.5, 6.4)
**8192**	6.78	7.08	6.28	1.63	1.60	1.64	(6.3, 7.2)	(6.6, 7.5)	(5.8, 6.7)

**Table 2:** Superset Search number of hops.

Nodes Number	Average			Standard Deviation			Confidence Interval (95%)		
	100	1000	10,000	100	1000	10,000	100	1000	10,000
**128**	18.28	4.54	3.52	8.44	1.54	1.19	(15.9, 20.6)	(4.1, 4.9)	(3.1, 3.8)
**256**	35.90	6.80	4.16	17.89	2.25	1.43	(30.9, 40.8)	(6.1, 7.4)	(3.7, 4.5)
**512**	51.18	12.16	4.46	37.85	3.29	1.31	(40.6, 61.6)	(11.2, 13.0)	(4.1, 4.8)
**1024**	91.06	21.70	5.08	72.44	6.23	1.68	(70, 111)	(19.9, 23.4)	(4.6, 5.5)
**2048**	115.70	34.56	7.84	98.39	13.00	1.98	(88, 142)	(30.9, 38.1)	(7.2, 8.3)
**4096**	196.00	63.38	11.92	186.88	25.37	2.64	(144, 247)	(56.3, 70.4)	(11.1, 12.6)
**8192**	243.90	120.38	20.38	253.59	68.65	6.28	(173, 314)	(101, 139)	(18.6, 22.1)

**Table 3:** Average response latency and confidence interval for the Create KFrags and Get CFrags operations in a round, varying *t* and *k*.

*k*	*t*	Create KFrags (ms)		Get CFrags (ms)	
		Average	Conf Int (95%)	Average	Conf Int (95%)
**10**	**1**	75.6	(72.11, 79.09)	106.63	(104.79, 108.47)
	**2**	86.58	(82.07, 91.09)	116.01	(113.49, 118.54)
	**3**	88.23	(82.38, 94.09)	120.17	(117.04, 123.3)
	**4**	100.48	(94.42, 106.53)	127.98	(124.23, 131.73)
**20**	**1**	155.96	(144.22, 167.69)	128.38	(122.94, 133.82)
	**2**	130.32	(122.91, 137.73)	135.27	(130.74, 139.79)
	**3**	144.0	(136.01, 152.0)	152.28	(146.56, 157.99)
	**4**	146.92	(135.99, 157.85)	163.61	(154.72, 172.49)
**30**	**1**	113.11	(107.89, 118.33)	119.94	(116.93, 122.95)
	**2**	146.23	(140.54, 151.92)	141.16	(137.83, 144.49)
	**3**	172.57	(163.51, 181.62)	167.19	(160.77, 173.62)
	**4**	162.65	(154.41, 170.89)	173.43	(167.14, 179.73)
**40**	**1**	211.23	(200.45, 222.01)	158.86	(152.58, 165.15)
	**2**	176.49	(168.25, 184.73)	166.48	(160.42, 172.53)
	**3**	206.08	(196.19, 215.97)	192.9	(185.59, 200.22)
	**4**	220.54	(210.67, 230.4)	209.77	(202.55, 216.98)
**50**	**1**	122.28	(117.61, 126.95)	122.32	(119.94, 124.7)
	**2**	189.77	(179.35, 200.2)	170.66	(163.35, 177.96)
	**3**	235.03	(224.69, 245.36)	215.84	(207.61, 224.08)
	**4**	267.82	(257.65, 277.99)	251.73	(243.17, 260.3)
**60**	**1**	172.14	(166.32, 177.95)	148.48	(144.76, 152.19)
	**2**	177.44	(169.55, 185.34)	172.77	(166.75, 178.8)
	**3**	225.4	(216.35, 234.45)	208.26	(201.29, 215.22)
	**4**	140.75	(135.36, 146.15)	159.98	(155.94, 164.03)
**70**	**1**	158.52	(152.33, 164.7)	141.2	(137.57, 144.83)
	**2**	179.65	(173.0, 186.3)	166.32	(161.58, 171.05)
	**3**	275.55	(264.45, 286.65)	250.54	(241.68, 259.4)
	**4**	230.97	(221.41, 240.53)	229.48	(221.51, 237.45)
**80**	**1**	178.65	(172.19, 185.1)	153.97	(149.92, 158.02)
	**2**	198.21	(190.55, 205.88)	178.61	(173.34, 183.89)
	**3**	204.39	(196.89, 211.89)	205.24	(198.95, 211.53)
	**4**	226.86	(217.05, 236.66)	231.71	(223.5, 239.92)

**Table 4:** *k*-DaO smart contract methods’ gas usage. Results are indicative and can change on the basis of the input data.

Smart Contract	Method	Gas Usage
**DataOwnerContract**	grantAccess()	96,436
	requestAccess()	142,648
	grantAccessRequest()	77,706
	revokeAccess()	30,126
**AggregatorContract**	requestAccessToData()	698,854
	createkDaO()	447,958
**kDaO**	submitProposal()	133,501
	submitRefundProposal()	362,489
	submitSuggestion()	114,523
	vote()	188,539
	changeVote()	153,587
	executeRefundProposal()	82,672
**kDaOToken**	transfer()	52,311
**TokenTimelockProxy**	lockTokens()	246,525
**TokenTimelockUpgreadeable**	release()	45,808

## Data Availability

The complete dataset and the reference software referenced in the performance evaluation are stored in [77,82,84], following the FAIR data principles for access and reuse of models [86].

## Abbreviations

The following abbreviations are used in this manuscript:

API	Application Programming Interface
ACL	Access Control List
CFrag	Capsule Fragment
CID	Content Identifier
DAG	Directed Acyclic Graph
DAO	Decentralized Autonomous Organization
DFS	Decentralized File Storage
DHT	Distributed Hash Table
DLT	Distributed Ledger Technology
GDPR	General Data Protection Regulation
IBFT	Istanbul Byzantine Fault-Tolerant
IPFS	InterPlanetary File System
KFrag	Key Fragment
P2P	Peer-to-Peer
PIMS	Personal Information Management System
PDS	Personal Data Store
PRE	Proxy Re-Encryption
SSI	Self-Sovereign Identity
TPRE	Threshold Proxy Re-Encryption
UML	Universal Modeling Language
W3C	World Wide Web Consortium
